# Urinary nighttime and first morning cortisol levels in patients with Prolonged Grief Disorder (PGD) and healthy controls: early outcomes

**DOI:** 10.1192/j.eurpsy.2025.1696

**Published:** 2025-08-26

**Authors:** V. Pedrinelli, V. Dell’Oste, S. Fantasia, A. Bordacchini, B. Rimoldi, L. Parrini, D. Andreoli, D. Gravina, L. Palego, G. Giannaccini, L. Betti, C. Carmassi

**Affiliations:** 1 University of Pisa, Pisa; 2 University of Siena, Siena, Italy

## Abstract

**Introduction:**

Prolonged Grief Disorder (PGD) has been recently included in the “Trauma and Stressor-Related Disorders” chapter of the latest edition of the DSM (DSM-5-TR), being fully acknowledged among mental disorders. PGD extend the period of acute grief and increase the risk for a wide range of health impairments. The availability of biomarkers for mental disorders is thought to be crucial in the development of precision psychiatry. The hypothalamus-pituitary-adrenal (HPA) axis activity and cortisol reactivity have frequently been investigated in mental disorders. Data on neurobiology of PGD is lacking. Some studies found that PGD might be associated with increased HPA axis activity and impaired autonomic nervous system regulation.

**Objectives:**

Aim of the present study was to examine the levels of cortisol excreted in urine during the night and first morning and to assess any differences and specificity of HPA axis functioning in a group of individuals with PGD and in one of healthy controls.

**Methods:**

Thirthy-three subjects, comprising 16 subjects diagnosed with PGD (PGD group) and 17 controls (CTL group), were recruited at the Psychiatric Clinic of the University of Pisa (Pisa, Italy). Psychometric assessments included: the Structured Clinical Interview for Mental Disorders-Clinician Version (SCID-5-CV), the Inventory of Complicated Grief (ICG) and the Impact of Events Scale-Revised (IES-R). Enrolled subjects, previously informed on collection procedures, delivered urine samples to the health care providers the same day of the clinical evaluation. Urine cortisol levels were measured by indirect enzyme-linked immunosorbent assays (ELISAs). Analyses were carried out at the Department of Pharmacy of the University of Pisa. Between-groups differences were performed by the non-parametric Mann-Whitney (MW). A *p- *value <.05 was considered statistically significant.

**Results:**

Descriptive results showed a higher variability (SDs and interquartile ranges) of urinary cortisol levels (total

*μ*g) in the PGD group in respect to the CTL one; by inferential statistics, MW comparisons showed significantly higher urinary cortisol levels in PGD group vs CTL one (*p*< .05).

**Conclusions:**

Results report that PGD patients had impaired cortisol outputs with respect to control subjects, suggesting a different pattern of production of the hormone during the night and the sleep-wake shift. If this prelimary data will be confirmed in wider samples, there will be a need to understand whether the increased cortisol profile reported in PGD may be due to increased production of the hormone at night (sleep alterations), to an increased peak on awakening (hyperarousal) or both conditions. Such findings might help to define more accurate patient-tailored therapeutic interventions.

**Disclosure of Interest:**

None Declared

